# G-formula with multiple imputation for causal inference with incomplete data

**DOI:** 10.1177/09622802251316971

**Published:** 2025-03-31

**Authors:** Jonathan W Bartlett, Camila Olarte Parra, Emily Granger, Ruth H Keogh, Erik W van Zwet, Rhian M Daniel

**Affiliations:** 1 Department of Medical Statistics, 4906 London School of Hygiene & Tropical Medicine, London, UK; 2 Department of Biomedical Data Sciences, Leiden University, Leiden, the Netherlands; 3 Division of Population Medicine, 2112 Cardiff University, Cardiff, UK

**Keywords:** G-formula, multiple imputation, synthetic imputation

## Abstract

G-formula is a popular approach for estimating the effects of time-varying treatments or exposures from longitudinal data. G-formula is typically implemented using Monte-Carlo simulation, with non-parametric bootstrapping used for inference. In longitudinal data settings missing data are a common issue, which are often handled using multiple imputation, but it is unclear how G-formula and multiple imputation should be combined. We show how G-formula can be implemented using Bayesian multiple imputation methods for synthetic data, and that by doing so, we can impute missing data and simulate the counterfactuals of interest within a single coherent approach. We describe how this can be achieved using standard multiple imputation software and explore its performance using a simulation study and an application from cystic fibrosis.

## 1. Introduction

The collection of methods referred to as G-methods, developed by James Robins and co-workers, can provide valid inference for the effects of time-varying exposures or treatments in the presence of time-varying confounders – variables that affect treatment over time and the outcome of interest – even when these are affected by previous values of treatment.^1^ One such method is parametric G-formula (sometimes known as G-computation). Parametric G-formula involves postulating models for the time-varying confounders and outcomes. The expected outcome under specified longitudinal treatment regimes of interest can then be estimated and contrasted. The evaluation of G-formula estimators generally involves intractable integrals. To overcome this in practice, G-formula implementations make use of Monte-Carlo integration, in which counterfactual outcomes are simulated under the treatment regimes of interest.^2,3^ Inference for Monte-Carlo based G-formula estimators is typically performed using bootstrapping.

A common complication in this context is missing values in the time-varying confounders, treatment and/or outcome variables. Existing implementations of G-formula in statistical software either impute such missing values once before fitting the required models^2^ or fit the required models discarding person-visit observations which contain missing values in the variables concerned.^3^ Alternatively, analysts may opt for ad-hoc approaches such as last observation carried forward to handle such missingness, which may make implausible assumptions about missing data.^4^ In contrast, multiple imputation (MI) of missing values is often nowadays considered an attractive approach to handling missing data, since it makes efficient use of the observed data and may in some situations make a more plausible assumption about missing data than discarding incomplete observations.^5^ In principle G-formula can be used after MI: The G-formula method is applied to each imputed dataset, using bootstrapping to obtain the within-imputation variance estimate, and the estimates are then pooled using Rubin’s rules. The drawback to this approach is a very high computational cost, because the G-formula approach with bootstrapping, which itself is computationally expensive, must be repeated M times, where M is the chosen number of imputations. To address this, we show how G-formula for estimating causal effects can be combined with MI to handle missing data in such a way that the computationl burden is much reduced. Specifically, we show that methods for adapting MI for missing data to generate synthetic datasets can be used to implement a G-formula estimator.^6^ As such, we show how MI methods can be used to impute both missing actual data and missing counterfactuals of interest in a single coherent approach. Moreover, we show that a simple variance combination rule previously derived for use with synthetic MI can be used to estimate the variance of the resulting G-formula MI estimator, obviating the need for bootstrapping.

This paper is organised as follows. In Section 2 we review parametric G-formula and how it is typically implemented. In Section 3 we describe how G-formula can be implemented by exploiting existing methodology for using MI to generate synthetic datasets. In Section 4 we report the results of simulation studies investigating the performance of this approach. Section 5 describes results of an illustrative analysis of data from a cystic fibrosis registry. We conclude in Section 6 with a discussion.

## 2. Review of G-formula

**Figure 1.**
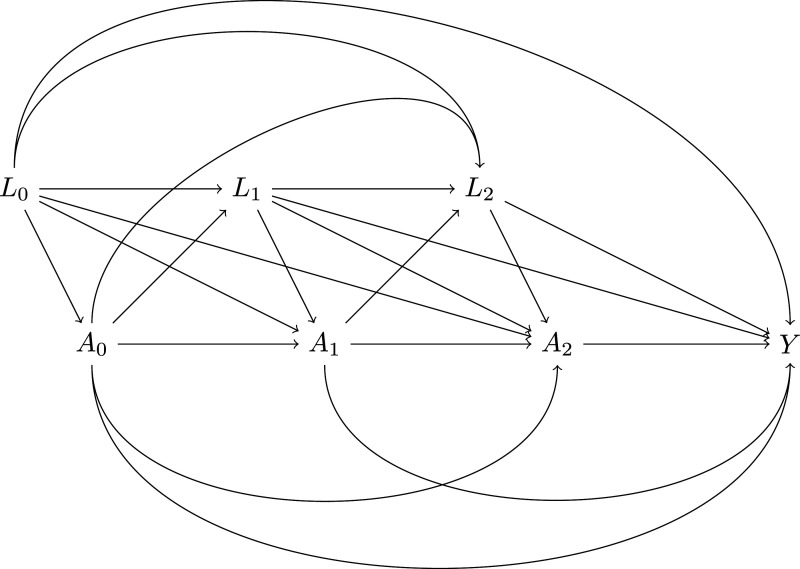
Directed acyclic graph (DAG) of a study with time-varying treatments $A_{0},A_{1},A_{2}$ , time-varying confounders $L_{0},L_{1},L_{2}$ and final outcome Y .

Suppose we have a sample of data from $n_{\text{obs}}$ independent individuals from some well-defined population. For individual i we collect confounder measurements $L_{it}$ at times t=0,1,…,T . We also collect measurements on the treatment $A_{it}$ at each time, and a final outcome $Y_{i}$ . Note that in some settings (e.g. our example in Section 5) earlier measurements of the outcome variable may be included in the time-varying confounders $L_{it}$ . For concreteness, we consider in the following the case of T=2 , although the developments naturally extend to T>2 . Figure 1 shows a directed acyclic graph (DAG) depicting the assumed causal structure between the variables. We also suppose for now that $L_{it}$ is a real-valued scalar, but natural adaptations/extensions to discrete and higher-dimensional confounders apply. Let $Y^{\bar{a}}$ denote the potential outcome for an individual when their treatment sequence has been set to a specific value $\bar{a}$ , e.g. (1,1,1) . G-formula relies on certain identification assumptions being satisfied, for the details of which we refer the reader to Chapter 19 of Hernán and Robins.^7^

The G-formula estimator of $\mu =E(Y^{\bar{a}})=E(Y^{a_{0},a_{1},a_{2}})$ for specified values of $a_{0}$ , $a_{1}$ and $a_{2}$ is then based on the fact that under the aforementioned identifying assumptions

(1) $$\begin{array}{r} E(Y^{\bar{a}})=\int_{l_{0}}\int_{l_{1}}\int_{l_{2}}E(Y|a_{0},a_{1},a_{2},l_{0},l_{1},l_{2})f(l_{2}|a_{0},a_{1},l_{0},l_{1})f(l_{1}|a_{0},l_{0})f(l_{0})dl_{2}dl_{1}dl_{0} \end{array}$$

To implement G-formula we specify and fit models

(2) $$\begin{array}{rl} & f(Y|A_{0},A_{1},A_{2},L_{0},L_{1},L_{2};\psi_{Y})\\ & f(L_{2}|A_{0},A_{1},L_{0},L_{1};\psi_{2})\\ & f(L_{1}|A_{0},L_{0};\psi_{1})\\ & f(L_{0};\psi_{0}) \end{array}$$

and, in principle, evaluate (1) replacing the unknown densities and expectation by their estimated counterparts. However, since (1) cannot generally be evaluated analytically, implementations of G-formula are typically based on Monte-Carlo integration, through simulation of the longitudinal confounders and outcome for each of $n_{\text{syn}}$ individuals, under the treatment combination(s) of interest. That is, given maximum likelihood estimates of the parameters in the conditional models in equation (2), denoted ${\hat{\psi }}_{0},{\hat{\psi }}_{1},{\hat{\psi }}_{2},{\hat{\psi }}_{Y}$ , we sequentially simulate for each individual $i=1,\ldots ,n_{\text{syn}}$ as follows

$$\begin{array}{rl} {\tilde{L}}_{i0} & ∼f(L_{0};{\hat{\psi }}_{0})\\ {\tilde{L}}_{i1} & ∼f(L_{1}|a_{0},{\tilde{L}}_{i0};{\hat{\psi }}_{1})\\ {\tilde{L}}_{i2} & ∼f(L_{2}|a_{0},a_{1},{\tilde{L}}_{i0},{\tilde{L}}_{i1};{\hat{\psi }}_{2})\\ {\tilde{Y}}_{i} & ∼f(Y|a_{0},a_{1},a_{2},{\tilde{L}}_{i0},{\tilde{L}}_{i1},{\tilde{L}}_{i2};{\hat{\psi }}_{Y}) \end{array}$$

The Monte-Carlo G-formula estimator of $E(Y^{a_{0},a_{1},a_{2}})$ is then $\frac{1}{n_{\text{syn}}}\sum_{i=1}^{n_{\text{syn}}}{\tilde{Y}}_{i}$ . The number of individuals to simulate for, $n_{\text{syn}}$ , could be set equal to $n_{\text{obs}}$ , but choosing a larger value reduces Monte-Carlo error in the estimator. For statistical inference, implementations of G-formula in Stata and R rely on the use of non-parametric bootstrapping,^2,3^ which as noted in Section 1, is computationally intensive. Note that here bootstrapping involves creating datasets by resampling data at the individual level (i.e. in wide data format), and repeating the estimation process on each of these.

While we have stated that a model $f(L_{0};\psi_{0})$ is specified and used, in fact this is not needed and is not typically used. Instead, a non-parametric model for $f(L_{0})$ is used, and the simulation is performed by sampling a value of $L_{0}$ from its empirical distribution (that is, sampling $n_{\text{syn}}$ times with replacement from the $n_{\text{obs}}$ observations of $L_{0}$ ). Moreover, when as is often the case interest lies in the mean $E(Y^{\bar{a}})$ (as opposed to some other function of the distribution of $Y^{\bar{a}}$ ), it suffices to specify a model for $E(Y|A_{0},A_{1},A_{2},L_{0},L_{1},L_{2})$ , rather than for the full conditional distribution $f(Y|A_{0},A_{1},A_{2},L_{0},L_{1},L_{2})$ . Our choice in the preceding to describe a version of G-formula that specifies the latter conditional distribution (rather than mean) model is motivated by the fact this version matches the approach taken in an MI implementation of G-formula, which we describe next.

## 3. G-formula via MI

In this section we describe how a Monte-Carlo G-formula estimator can be implemented using MI methods. In Section 3.1 we describe how the point estimator is constructed using MI. In Section 3.2 we explain why Rubin’s standard variance estimator is biased in this instance, and describe an alternative variance estimator, which was derived in the context of using MI to generate synthetic datasets by Raghunathan et al.^6^ In Section 3.3 we describe how standard MI software can be used to implement the approach. Lastly, in Section 3.4 we describe how the approach readily extends to accommodate missing actual data (as opposed to missing counterfactual data).

### 3.1. Point estimation

To estimate $E(Y^{\bar{a}})$ by MI, first augment the observed dataset by adding $n_{\text{syn}}$ additional rows. Let $n=n_{\text{obs}}+n_{\text{syn}}$ , such that n denotes the number of rows in the augmented dataset (i.e. the original plus augmented rows). In the augmented rows, as shown in Table 1, the baseline and time-varying confounders $\left(L_{0},\ldots ,L_{T}\right)$ and final outcome Y are set to missing, while the treatment variables are set to their values under the regime of interest, i.e. $A_{0}=a_{0},A_{1}=a_{1},\ldots ,A_{T}=a_{T}$ . The variable R indicates whether the data row was in the original sample ( R=1 ) or not ( R=0 ).

**Table 1.** G-formula via multiple imputation (MI) data setup.

R	$L_{0}$	$A_{0}$	$L_{1}$	$A_{1}$	$L_{2}$	$A_{2}$	Y
1	−0.3	0	0.5	0	2.2	1	1.3
1	2.3	1	4.2	1	4.6	1	5.5
1	−0.5	1	0.4	0	0.8	1	1.9
1	−0.1	0	1.6	1	4.1	0	7.0
1	0.4	1	1.9	1	3.5	1	6.2
0	NA	1	NA	1	NA	1	NA
0	NA	1	NA	1	NA	1	NA
0	NA	1	NA	1	NA	1	NA
0	NA	1	NA	1	NA	1	NA
0	NA	1	NA	1	NA	1	NA

The original dataset (top part) is augmented with additional rows (bottom part). In the augmented part, confounders $L_{0},L_{1},L_{2}$ and outcome Y are set to missing (indicated here by NA), while the treatment variables $A_{0},A_{1},A_{2}$ are set to their values under the regime of interest (here 1,1,1 ). The variable R indicates whether the row is originally observed data ( R=1 ) or not ( R=0 ).

Next, Bayesian MI is used to generate M imputations of the missing values in this augmented dataset, using the chosen sequential models (equation (2)). In particular, this means each imputed dataset is generated conditional on a draw from the posterior distribution of the models’ parameters. Next, within imputation m ( m=1,…,M ), calculate the mean of Y in the augmented rows ( R=0 ), yielding ${\hat{\mu }}_{m}=\frac{\sum_{i=1}^{n}(1-R_{i})Y_{i}^{m}}{\sum_{i=1}^{n}(1-R_{i})}$ , where $Y_{i}^{m}$ denotes the imputation of $Y_{i}$ in imputation m . The mean outcome under the treatment regime of interest, $\mu =E(Y^{\bar{a}})$ , is then estimated as $\hat{\mu }=\frac{1}{M}\sum_{m=1}^{M}{\hat{\mu }}_{m}$ .

The resulting estimator $\hat{\mu }$ differs from the Monte-Carlo G-formula estimator described in Section 2 in two respects. First, it generates multiple imputed datasets, analyses each, and combines the estimates, whereas the standard G-formula estimator estimates the mean based on one imputed dataset. Second, whereas the standard G-formula estimator generates the imputed values conditional on efficient (e.g. MLE) estimates of the parameters for the models in equation (2), as described above the G-formula MI approach generates each imputed dataset conditional on an imputation specific draw from the posterior distribution of the imputation model parameters. These differences are of no consequence for the probability limits of the two estimators – if we choose $n_{\text{syn}}=kn_{\text{obs}}$ for some fixed k , as $n_{\text{obs}}\to \infty$ , the theory for imputation estimators of Robins and Wang^8^ implies both estimators converge in probability to $\mu =E(Y^{\bar{a}})$ (provided the assumed models are correctly specified).

For the G-formula via MI approach we propose, the impact of generating each imputed dataset conditional on posterior draws of the model parameters, rather than an efficient observed data estimate, is to increase the asymptotic variance of the estimator, but this increase goes to zero as M→∞ .^9^ Moreover, this step is essential to facilitate straightforward variance estimation, which we describe next.

### 3.2. Variance estimation

The variance of an MI estimator is typically estimated using Rubin’s variance estimator $(1+M^{-1})\hat{B}+\hat{V}$ where $\hat{B}=\frac{1}{M-1}\sum_{m=1}^{M}({\hat{\mu }}_{m}-\hat{\mu }{)}^{2}$ denotes the between-imputation variance and $\hat{V}=\frac{1}{M}\sum_{m=1}^{M}\hat{\text{Var}}({\hat{\mu }}_{m})$ denotes the average within-imputation variance.^5^ Rubin’s variance estimator is valid when the imputation and model or procedure used to analyse the data are so-called congenial.^10^ When congeniality does not hold, Rubin’s variance estimator may (but not in all cases) be biased.^8,11^ One such situation where Rubin’s variance estimator is biased is when only a subset of the records used to fit the imputation model is used to fit the analysis model, of which the G-formula via MI estimator is one such example – the original observed dataset is used to fit the imputation models, while only the augmented dataset rows are used to fit the analysis model (estimating the mean of Y among those with R=0 ). As such we may anticipate that Rubin’s variance estimator will be biased for the G-formula via MI point estimator.

The G-formula via MI estimator is closely related to the use of MI to generate samples from synthetic populations, first proposed by Rubin.^12^ Here the objective is to release these synthetic samples rather than the original data in order to protect the confidentiality of survey respondents’ data. For synthetic MI, Raghunathan et al.^6^ developed ${\hat{V}}_{\text{syn}}=(1+M^{-1})\hat{B}-\hat{V}$ as an estimator of $\text{Var}(\hat{\mu })$ from both Bayesian and repeated sampling perspectives.

To build intuition for ${\hat{V}}_{\text{syn}}$ , we now show it is unbiased for $\text{Var}(\hat{\mu })$ in a highly simplified but instructive setting. Suppose we observe data from $n_{\text{obs}}$ individuals on an outcome $Y∼N(\mu ,\sigma^{2})$ and interest lies in inference for μ . Here to estimate the mean μ we can of course trivially use the sample mean $\bar{Y}=n_{\text{obs}}^{-1}\sum_{i=1}^{n_{\text{obs}}}Y_{i}$ , which has repeated sampling variance $\sigma^{2}/n_{\text{obs}}$ . Suppose however that we use Bayesian MI to generate M new imputed datasets of size $n_{\text{syn}}$ . For simplicity, we assume $\sigma^{2}$ is known. In this case, under the standard non-informative prior for μ , to generate imputation m we first draw ${\tilde{\mu }}_{\left(m\right)}∼N\left(\bar{Y},\frac{\sigma^{2}}{n_{\text{obs}}}\right)$ . For $i=n_{\text{obs}}+1,\ldots ,n$ we then simulate (impute) $n_{\text{syn}}$ new Y values $Y_{i(m)}={\tilde{\mu }}_{\left(m\right)}+\epsilon_{i(m)}$ , where $\epsilon_{i(m)}∼N(0,\sigma^{2})$ .

Having generated imputed/synthetic datasets for m=1,…,M , the estimate of μ based on them is then

$$\begin{array}{rl} \hat{\mu } & =\frac{1}{M}\sum_{m=1}^{M}{\hat{\mu }}_{m}\\ & =\frac{1}{M}\sum_{m=1}^{M}\frac{1}{n_{\text{syn}}}\sum_{i=n_{\text{obs}}+1}^{n}\left\{{\tilde{\mu }}_{\left(m\right)}+\epsilon_{i(m)}\right\}\\ & =\frac{1}{M}\sum_{m=1}^{M}{\tilde{\mu }}_{\left(m\right)}+\frac{1}{n_{\text{syn}}M}\sum_{m=1}^{M}\sum_{i=n_{\text{obs}}+1}^{n}\epsilon_{i(m)} \end{array}$$

Letting $\tilde{\mu }=\{{\tilde{\mu }}_{\left(1\right)},\ldots ,{\tilde{\mu }}_{\left(M\right)}\}$ , this has variance

$$\begin{array}{rl} \text{Var}(\hat{\mu }) & =E\left\{\text{Var}(\hat{\mu }|\tilde{\mu })\right\}+\text{Var}\left\{E(\hat{\mu }|\tilde{\mu })\right\}\\ & =E\left\{\text{Var}\left(\frac{1}{n_{\text{syn}}M}\sum_{m=1}^{M}\sum_{i=n_{\text{obs}}+1}^{n}\epsilon_{i(m)}|\tilde{\mu }\right)\right\}+\text{Var}\left\{\frac{1}{M}\sum_{m=1}^{M}{\tilde{\mu }}_{\left(m\right)}\right\}\\ & =\frac{\sigma^{2}}{n_{\text{syn}}M}+\text{Var}\left\{E\left(\frac{1}{M}\sum_{m=1}^{M}{\tilde{\mu }}_{\left(m\right)}|\bar{Y}\right)\right\}+E\left\{\text{Var}\left(\frac{1}{M}\sum_{m=1}^{M}{\tilde{\mu }}_{\left(m\right)}|\bar{Y}\right)\right\}\\ & =\frac{\sigma^{2}}{n_{\text{syn}}M}+\text{Var}(\bar{Y})+E\left(\frac{\sigma^{2}/n_{\text{obs}}}{M}\right)\\ & =\frac{\sigma^{2}}{n_{\text{syn}}M}+\left(1+M^{-1}\right)\frac{\sigma^{2}}{n_{\text{obs}}} \end{array}$$

With $\sigma^{2}$ known, the within-imputation variance is $\sigma^{2}/n_{\text{syn}}$ for every imputed dataset, and so $\hat{V}=\sigma^{2}/n_{\text{syn}}$ . Conditional on the observed data $\bar{Y}$ , the between-imputation variance estimator $\hat{B}$ is an unbiased estimator of

$$\begin{array}{rl} \text{Var}({\hat{\mu }}_{m}|\bar{Y}) & =\text{Var}\left({\tilde{\mu }}_{\left(m\right)}+\frac{1}{n_{\text{syn}}}\sum_{i=n_{\text{obs}}+1}^{n}\epsilon_{i(m)}|\bar{Y}\right)\\ & =\frac{\sigma^{2}}{n_{\text{obs}}}+\frac{\sigma^{2}}{n_{\text{syn}}} \end{array}$$

Thus, unlike in the missing data setting, the between-imputation variance captures variability both due to uncertainty about μ in the observed data estimate and the additional variability due to effectively taking new random samples of size $n_{\text{syn}}$ from the population for each imputation.^13^ In particular, this means that as noted earlier, Rubin’s usual variance estimator is biased upwards for $\text{Var}(\hat{\mu })$ , except in the case that $n_{\text{syn}}\to \infty$ , in which case the within-imputation variance $\hat{V}$ goes to zero. In contrast, the expected value of ${\hat{V}}_{\text{syn}}$ is

$$\begin{array}{rl} E({\hat{V}}_{\text{syn}}) & =E\{(1+M^{-1})\hat{B}-\hat{V}\}=(1+M^{-1})\left(\frac{\sigma^{2}}{n_{\text{obs}}}+\frac{\sigma^{2}}{n_{\text{syn}}}\right)-\frac{\sigma^{2}}{n_{\text{syn}}}\\ & =\frac{\sigma^{2}}{n_{\text{syn}}M}+(1+M^{-1})\frac{\sigma^{2}}{n_{\text{obs}}}\\ & =\text{Var}(\hat{\mu }) \end{array}$$

such that ${\hat{V}}_{\text{syn}}$ is unbiased for $\text{Var}(\hat{\mu })$ . In Appendix A of the Supplemental material we use the results of Robins and Wang^8^ for the asymptotic behaviour of Rubin’s variance estimator to justify ${\hat{V}}_{\text{syn}}$ for the G-formula MI approach we have described.

As noted by Reiter^14^ and Raghunathan et al.,^6^ the variance estimator ${\hat{V}}_{\text{syn}}$ can be negative. In the simplified normal mean example, we show in Appendix B of the Supplemental material that the probability of this occurring is approximately given by $P\left\{\chi_{M-1}^{2}<\frac{M}{\frac{n_{\text{syn}}}{n_{\text{obs}}}+1}\right\}$ . Consideration of this shows, in line with the results of Reiter,^14^ that the probability of a negative variance estimate can be made arbitrarily small by increasing M and/or $n_{\text{syn}}$ . Reiter^14^ considered how the latter can be chosen using some initial synthetic imputations to ensure the probability that ${\hat{V}}_{\text{syn}}$ is negative is sufficiently small. In Section 4 we investigate the performance of a procedure where if ${\hat{V}}_{\text{syn}}\leq 0$ , we successively add additional batches of M imputations until ${\hat{V}}_{\text{syn}}>0$ . To account for the impact of using a finite number of imputations M , Raghunathan and Rubin^15^ proposed inference based on a t -distribution with degrees of freedom $v_{f}=(M-1){\left(1-\frac{M\hat{V}}{(M+1)\hat{B}}\right)}^{2}$ , the performance of which we explore in simulations in Section 4.

### 3.3. Implementation using imputation software

To implement the proposed approach, as described previously, the observed dataset of size $n_{\text{obs}}$ is augmented by an additional $n_{\text{syn}}$ rows in which all variables are set to missing except the treatment variables, which are set to their values under the regime of interest, i.e. $A_{0}=a_{0},A_{1}=a_{1},\ldots ,A_{T}=a_{T}$ . MI software, such as the mice package in R, can then be applied to the resulting dataset, with options specified so that the time-varying confounders and outcome are imputed sequentially in time as per the models given in equation (2). Since the missingness pattern is monotone, no iterative methods such as Markov Chain Monte Carlo are required. Following imputation, the augmented subset is extracted from each imputed dataset, and the mean of Y is evaluated in each, yielding ${\hat{\mu }}_{m}$ ( m=1,…,M ), along with a corresponding complete data variance estimate. The variance estimator ${\hat{V}}_{\text{syn}}$ can then be evaluated.

Ordinarily interest focuses on the contrast of potential outcome means under two (or more) different treatment regimes. To estimate the corresponding contrast in potential outcome means, we augment the observed dataset twice. In the second augmentation part, the treatment variables are set according to the second treatment regime of interest. The difference in potential outcome means can be estimated by the difference in simulated outcomes between the two augmented parts. The variance of the resulting estimator can be estimated by the sum of the variance estimator ${\hat{V}}_{\text{syn}}$ when applied to the two regimes of interest, since the sets of synthetic imputations for the two regimes are independent (conditional on the parameter draws used to impute).

Implementation of the preceding steps using packages such as mice in R is relatively straightforward. Nonetheless, to facilitate use of the approach, we provide the R package gFormulaMI. This augments the supplied dataset as described above and imputes missing data using the mice package. The resulting imputed datasets contain only the augmented portion of the imputations (with R=0 ), which can be used to estimate potential outcome means and contrasts of these. The point estimates and variances from the analysis of these imputations are then passed to a function implementing the variance estimator ${\hat{V}}_{\text{syn}}$ .

As noted earlier, the standard (non-Bayesian) implementation of G-formula avoids specification of a model for $f(L_{0})$ , and instead simulates from the empirical distribution of $L_{0}$ . This has the advantage of saving the analyst from concerns about misspecification of a model for $L_{0}$ . In the context of MI for generation of synthetic samples, Raghunathan et al.^6^ proposed using the approximate Bayesian bootstrap approach of Rubin and Schenker.^16^ In Section 4.1 we investigate in simulations the performance of using this approach for Bayesian non-parametric imputation of $L_{0}$ .

### 3.4. Missing data

Now suppose that there are some data missing which we want to handle by MI. Missing data could occur in either the longitudinal confounders $L_{it}$ , the final outcome $Y_{i}$ , or the time-varying treatment variables $A_{it}$ . We suppose that the missing at random assumption is deemed plausible for the missing values. Consider again the augmented data formed by adding to the observed dataset (which itself now has some missing values) the additional rows corresponding to the treatment regime(s) of interest with all variables set to missing expect the treatment variables. Suppose we then generate M imputations of the missing values (missing actual and potential outcome values) in this augmented dataset from a Bayesian model. Then in Appendix A we argue why the theory of Wang and Robins^8^ continues to show that ${\hat{V}}_{syn}$ is a valid variance estimator for $\hat{\mu }$ .

To multiply impute the missing values in the original dataset and the missing potential outcomes, we propose a two-stage approach where first we generate M imputations of the missing values in the original data portion ( R=1 ). In the second stage, the missing potential outcomes in the augmented portion (R=0 ) are imputed conditional on the first stage imputed data (i.e. with the missing values in the R=1 rows imputed). In practice this approach can be implemented by first applying MI to the original dataset M times. The synthetic rows are then added to each of these, and the missing potential outcomes are then imputed once (in each of the M datasets) based on the sequential models (equation (2)).

Such a two-stage approach to impute missing values has been proposed previously for contexts where, as in the case here, there are two qualitatively different types of missing data.^17^ It can be justified when (a) the missing data (here missing actual data and missing potential outcomes) are MAR, and (b) the process that divides the missing values into the two parts does not depend on the missing data.^17^ The first condition follows since the missingness in the original data portion is assumed MAR and in the augmented part it is MCAR by design. The second condition holds because the division of missing data is on the basis of R , which is fully observed by construction. As originally conceived,^18^ such a two-stage imputation approach usually involves imputing each of the imputations generated from the first stage multiple (say N times), yielding M×N imputations. However, as noted by Harel,^17^ choosing N=1 , as we propose, is perfectly valid in terms of generating draws from the predictive distribution of the missing data.

A two-stage approach to handle missingness in the observed data in the context of using MI to generate synthetic samples was recently developed by Yu et al.^19^ They proposed that conditional on each of the M imputations of the original data, N synthetic imputations (samples) are generated. The parameter of interest is estimated on each of these M×N synthetic samples and then their average is calculated as the point estimate. Yu et al. developed a variance estimator for the resulting estimator. This approach decomposes variation into between-imputation variation (due to missing actual data), between-synthesis variation, and within-imputation and synthesis variation. In contrast, if as we propose one simulates one synthetic sample per imputation of the missing values in the original dataset (i.e. taking N=1 ), the between imputation variance $\hat{B}$ captures the sum of these first two components. The variance estimator ${\hat{V}}_{\text{syn}}$ we use corresponds to that in the two-stage approach developed by Yu et al. after making what we believe is a necessary minor correction to their published formula, upon setting N=1 (in their paper, L=1 ).

The models required for G-formula given in equation (2) do not fully specify the joint distribution of all the variables under consideration, since they do not specify models for the treatment variables. The imputation models used to impute the missing data in the original dataset should ideally be compatible with those used to impute the augmented rows. One way to achieve this is to specify a full joint model for all the variables by, in addition to the models in equation (2), specifying models for the time-varying treatment variables. That is, for t=0,1,…T , we specify models $f(A_{it}|{\bar{A}}_{i(t-1)},{\bar{L}}_{it})$ , such as suitable logistic regression models if treatment is binary. While imputation from such a joint model is possible using Bayesian model software such as JAGS, imputation is more commonly performed using methods such as chained equations, as implemented in the popular R package mice. As such, in Section 4.2 we investigate performance when the models used to impute missing data are not strictly compatible with the models specified and used by G-formula (in equation (2)).

In the setting with missing data, our R package gFormulaMI takes as input a set of M imputed datasets, for example obtained using the mice package. It then augments each imputed dataset with the required additional rows and imputes each dataset once.

## 4. Simulations

In this section we report the results of simulations performed to examine the empirical performance of the G-formula via MI approach. We first consider, in Section 4.1, the setting where there is no missing data. Next, in Section 4.2, we consider the situation where some data are missing.

### 4.1. No missing data

We simulated datasets for $n_{\text{obs}}=500$ individuals with a single continuous confounder L measured at times t=0,1,2 , corresponding binary treatments A , and a continuous final outcome Y . The specific data generating mechanism is given in Appendix C of the Supplemental material. We report results for estimates of $E(Y^{1,1,1})-E(Y^{0,0,0})$ , whose true value under the data generating mechanism is 3. The G-formula via MI approach was implemented using the mice package in R, imputing $L_{0}$ , $L_{1}$ , $L_{2}$ and Y from normal linear models including all the preceding (in time) treatment and confounder variables linearly. Since the missingness pattern is monotone, we specified that mice only perform one iteration. We investigated how performance varied with $n_{\text{syn}}$ and M , using values $n_{\text{syn}}=kn_{\text{obs}}$ for k=1,2,5,10 and M=5,10,25,50,100 . If in a particular simulation ${\hat{V}}_{\text{syn}}<0$ , we added an additional M imputations and re-calculated ${\hat{V}}_{\text{syn}}$ . This was repeated until ${\hat{V}}_{\text{syn}}>0$ .

Table 2 shows results based on 10,000 simulations per scenario. As expected since the imputation models were correctly specified, the G-formula via MI estimator for $E(Y^{1,1,1})-E(Y^{0,0,0})$ was unbiased for all values of M and $n_{\text{syn}}$ . The variance estimator ${\hat{V}}_{\text{syn}}$ was also essentially unbiased across all the scenarios. Confidence intervals calculated based on a t-distribution with degrees of freedom $v_{f}$ showed overcoverage for M=5 and M=10 , although as $n_{\text{syn}}$ increased this overcoverage diminished. For M=25,50,100 coverage was close to the nominal level. Confidence intervals calculated based on a standard normal showed substantial undercoverage for M=5 and M=10 , and this persisted even with larger values of $n_{\text{syn}}$ but coverage was close to nominal coverage for M=50 and M=100 . Lastly, when using a smaller initial value for M , sometimes additional imputations were required to ensure ${\hat{V}}_{\text{syn}}>0$ , as indicated by the mean and maximum M values in Table 2. However, the need for additional imputations reduced as $n_{\text{syn}}$ was increased: for $n_{\text{syn}}\geq 1000$ no additional imputations were needed when the initial number of imputations was 25 or higher.

**Table 2.** Simulation results for G-formula via multiple imputation ( MI) without any missing data.

M	Bias	Emp. SE	Est. SE	Raghu df 95% CI	Z 95% CI	Mean M	Max M
$n_{\text{syn}}=n_{\text{obs}}=500$							
5	−0.002	0.242	0.238	99.9	87.1	5.6	15
10	−0.001	0.229	0.224	98.4	89.9	10.2	20
25	0.000	0.225	0.220	95.1	92.7	25.0	50
50	−0.003	0.220	0.220	95.2	94.1	50.0	50
100	0.001	0.219	0.219	95.1	94.6	100.0	100
$n_{\text{syn}}=2n_{\text{obs}}=1000$							
5	0.000	0.241	0.229	99.4	87.0	5.2	15
10	0.000	0.232	0.222	96.2	90.2	10.0	20
25	0.001	0.223	0.220	95.2	93.4	25.0	25
50	0.001	0.223	0.220	94.8	94.0	50.0	50
100	−0.004	0.217	0.219	95.0	94.7	100.0	100
$n_{\text{syn}}=5n_{\text{obs}}=2500$							
5	0.001	0.242	0.227	97.9	87.3	5.0	10
10	−0.001	0.231	0.222	95.0	91.1	10.0	20
25	0.000	0.226	0.220	94.5	93.2	25.0	25
50	0.003	0.220	0.220	94.9	94.2	50.0	50
100	−0.002	0.218	0.219	95.0	94.7	100.0	100
$n_{\text{syn}}=10n_{\text{obs}}=5000$							
5	0.002	0.240	0.224	96.6	87.7	5.0	10
10	0.001	0.231	0.223	95.4	91.8	10.0	10
25	−0.001	0.221	0.220	95.2	94.0	25.0	25
50	0.000	0.222	0.219	94.9	94.3	50.0	50
100	0.003	0.219	0.219	95.4	95.1	100.0	100

Results are shown for different numbers of initial imputations M . Emp SE. gives the empirical standard error of the point estimates while Est. SE gives the mean estimated standard error based on ${\hat{V}}_{\text{syn}}$ . Raghu CI gives the coverage of t-based 95% confidence intervals based on the degrees of freedom $v_{f}$ while Z CI gives coverage for 95% confidence intervals constructed using N(0,1) quantiles. Mean M and Max M give the mean and maximum value of M required across the simulations in order to obtain ${\hat{V}}_{\text{syn}}>0$ .

For comparison with performance of G-formula based on the usual implementation approach, Table 3 shows results from 10,000 simulations under the same data generating mechanism obtained using the gfoRmula package. Here pooled models are fitted to the data in long form, as opposed to in wide form in our G-formula via MI implementation using mice. The results shown in Table 3 are based on assuming a normal linear model for the single continuous time-varying confounder, and included the past measurements of treatment and the confounder, plus visit time, as covariates. The results show the estimates were also unbiased, and had empirical SE very slightly below that achieved by the G-formula via MI approach when using M=100 imputations. Due to the computational burden of bootstrapping, we did not calculate bootstrap confidence intervals in these simulations, although there is no reason to expect them not to achieve nominal coverage here.

**Table 3.** Simulation results for G-formula using gfoRmula package, based on Monte-Carlo sample sizes of nsimul=500,1000,2500,5000 .

nsimul	Bias	Emp. SE
500	−0.003	0.218
1000	−0.003	0.215
2500	−0.006	0.217
5000	−0.006	0.217

Est. SE gives the mean estimated standard error based on ${\hat{V}}_{\text{syn}}$ .

Table 4 shows results of simulations for G-formula via MI performed using a smaller sample size of $n_{\text{obs}}=100$ . As expected, the empirical and estimated SEs were all larger. Otherwise, the results in terms of bias, coverage of confidence intervals, and requirement to add additional imputations to give a positive variance estimate were very similar to those with $n_{\text{obs}}=500$ shown in Table 2.

**Table 4.** Simulation results for G-formula via MI without any missing data, but using $n_{\text{obs}}=100$ .

M	Bias	Emp. SE	Est. SE	Raghu df 95% CI	Z 95% CI	Mean M	Max M
$n_{\text{syn}}=n_{\text{obs}}=100$							
5	0.000	0.554	0.547	99.8	87.7	5.6	15
10	−0.004	0.534	0.516	98.4	89.8	10.2	20
25	−0.002	0.508	0.507	95.9	93.4	25.0	50
50	−0.003	0.509	0.505	95.0	93.9	50.0	50
100	0.001	0.490	0.504	95.7	95.2	100.0	100
$n_{\text{syn}}=2n_{\text{obs}}=200$							
5	0.004	0.549	0.526	99.4	87.4	5.2	10
10	−0.003	0.525	0.510	96.3	90.5	10.0	20
25	0.000	0.513	0.508	94.9	93.4	25.0	25
50	−0.001	0.500	0.504	95.4	94.6	50.0	50
100	−0.007	0.497	0.506	95.3	95.0	100.0	100
$n_{\text{syn}}=5n_{\text{obs}}=500$							
5	−0.001	0.549	0.517	97.7	87.2	5.0	10
10	−0.004	0.523	0.512	95.7	91.3	10.0	20
25	−0.002	0.500	0.508	95.4	94.1	25.0	25
50	0.009	0.505	0.506	94.8	94.1	50.0	50
100	−0.004	0.502	0.504	94.8	94.6	100.0	100
$n_{\text{syn}}=10n_{\text{obs}}=1000$							
5	−0.002	0.548	0.520	96.6	87.7	5.0	10
10	0.001	0.527	0.512	95.1	91.4	10.0	10
25	−0.004	0.507	0.509	94.9	93.7	25.0	25
50	−0.002	0.502	0.506	95.4	95.0	50.0	50
100	−0.003	0.494	0.504	95.3	95.0	100.0	100

MI: multiple imputation; Emp SE.: empirical standard error of the point estimates; Est. SE: mean estimated standard error; Raghu CI: coverage of t-based 95% confidence intervals based on the degrees of freedom $v_{f}$ ; Z CI: coverage for 95% confidence intervals; Mean M : mean value of M ; Max M : maximum value of M .

We additionally ran 10,000 simulations with $M=50,n_{\text{obs}}=n_{\text{syn}}=500$ , using the approximate Bayesian bootstrap to impute $L_{0}$ . The variance estimator ${\hat{V}}_{\text{syn}}$ was again unbiased. The coverage of the confidence interval constructed using a t -distribution with degrees of freedom $v_{f}$ was 95.3% while the normal based confidence interval had coverage 94.3%, matching closely the corresponding results in Table 2.

### 4.2. Missing data

Next we performed simulations where some data were missing. Data in each of $L_{1}$ , $A_{1}$ , $L_{2}$ , $A_{2}$ and Y were made missing completely at random, with the probability of each being missing set to π , with π={0.05,0.1,0.25,0.5} . As such, the probability of an individual having complete data was $(1-\pi {)}^{5}$ and the average number of variables missing per individual was 5π . Thus π=0.5 is a really quite extreme scenario, with only approximately 3% of individuals having complete data.

To implement G-formula via MI we used an initial call to mice to impute the missing values M=50 times. The continuous variables $L_{1}$ , $L_{2}$ and Y were imputed using normal linear models while $A_{1}$ and $A_{2}$ were imputed using logistic regression models. Since the missingness pattern was not monotone, as per the standard chained equations algorithm, for imputation of a given variable, all the other variables were included as covariates. The number of iterations was left at its default value of 5, except for π=0.5 . Here, with a very large amount of missingness, we found that 50 iterations were required to achieve convergence. Having imputed the missing data, the additional $n_{\text{syn}}=500$ rows were added to each imputed dataset, and mice was applied to each of the M=50 datasets, specifying to impute using one iteration sequentially according to time, as used in the scenario without missing data.

Table 5 shows the results based on 10,000 simulations per value of π . The G-formula via MI estimator had minimal bias across all four scenarios. As we would expect, the empirical standard error increased with increasing amounts of missing data. The variance estimator ${\hat{V}}_{\text{syn}}$ was positive in all simulations and for all values of π when using an initial value of M=50 . ${\hat{V}}_{\text{syn}}$ was unbiased for the empirical SE. Confidence intervals based on a t-distribution with degrees of freedom $v_{f}$ showed slight overcoverage, while the normal based intervals showed slight undercoverage.

**Table 5.** Simulation results for G-formula via MI with missing data. π is the probability that each of $L_{1}$ , $A_{1}$ , $L_{2}$ , $A_{2}$ and Y are missing.

Scenario	π	Bias	Emp. SE	Mean est. SE	Raghu df 95% CI	Z 95% CI
1	0.05	0.000	0.226	0.225	94.9	93.8
2	0.10	−0.005	0.232	0.232	95.2	94.2
3	0.25	−0.010	0.260	0.258	95.0	94.1
4	0.50	−0.013	0.357	0.361	95.3	94.5

MI: multiple imputation; Emp SE.: empirical standard error of the point estimates; Est. SE: mean estimated standard error; Raghu CI: coverage of t-based 95% confidence intervals based on the degrees of freedom $v_{f}$ ; Z CI: coverage for 95% confidence intervals; Mean M : mean value of M ; Max M : maximum value of M .

## 5. Illustrative example

In this section we provide an illustrative example of the use of the G-formula via MI approach to investigate the effects of multiple treatments on lung function in people with cystic fibrosis (CF). Many people with CF are prescribed at least one mucoactive treatment to help improve lung function. In the UK, the most commonly prescribed nebulised mucoactive treatment is dornase alfa (DNase), and many patients already using DNase may later add or switch to hypertonic saline. Existing research investigates the effects of taking DNase or hypertonic saline alone, but the effects of using both treatments in combination are less well understood. Here we investigate the following question: for people with CF who are already established on DNase, does adding hypertonic saline have any additional benefit for lung function? In a recent study this question was investigated using marginal structural models estimated using inverse probability of treatment weighting to address time-dependent confounding.^20^

Our example uses data from the UK Cystic Fibrosis Registry, which collects longitudinal data on almost all people with CF in the UK.^21^ Longitudinal data are collected annually, when CF patients are seen at an outpatient clinic for a comprehensive review. The review data includes evaluation of clinical status, lung function, chronic medications, hospital admissions and health complications.

Using data from 2007 to 2018, we included individuals with CF, aged 6 years or older, who had been prescribed DNase, but not hypertonic saline, for at least two consecutive years. Organ transplant recipients, and people prescribed certain treatments (mannitol, ivacaftor, lumacaftor/ivacaftor, tezacaftor/ivacaftor) were excluded. Time zero was defined as the date of the most recent annual review at which the inclusion and exclusion criteria were met, but which allowed for the maximum possible follow-up time up to 5 years. The outcome of interest is lung function, and this is measured at the annual review as forced expiratory volume in one second (FEV ${}_{1}$ %). We estimate the mean differences in FEV ${}_{1}$ % at times 1–5 years had all individuals been prescribed DNase and hypertonic saline, compared to if all individuals were prescribed DNase only. The following variables were considered as confounders: sex, CFTR genotype, ethnicity, date of birth, rate of decline in FEV ${}_{1}$ % during the year prior to time 0, past FEV ${}_{1}$ %, respiratory infections, IV hospital admissions, BMI, pancreatic insufficiency and use of IV antibiotics. The first five of these were baseline confounders; the latter six were time-varying. Data may be missing if some information is not recorded at the annual review, or if the individual is no longer in the registry due to death or administrative end-of-follow-up. For the purposes of illustration, we assume all such missingness is at random.

Treatment effect estimates were obtained using the standard implementation of G-formula and using G-formula via MI. Standard implementation was done using the R package gfoRmula, with $n_{\text{syn}}$ set to 100,000 as used by De Stavola et al.^22^ Normal-based confidence intervals were constructed based on the non-parametric bootstrap estimated standard error (1000 bootstrap samples). Implementation of G-formula via MI was done using mice and gFormulaMI, with M=200 and $n_{\text{syn}}=n_{\text{obs}}=4,759$ . Since the G-formula MI estimator is the average of the estimates across the M imputations, this yields an effective Monte-Carlo sample size of 200×4759≈951,800 . Whereas gFormulaMI uses MI to handle missing data (as described in Section 3.4), the gfoRmula package handles missing data using a complete case analysis approach. For individuals who had any missing data at a particular time point, their data for that time point was not included in the conditional models used to simulate covariates and outcomes. Another difference between the two packages is the way the conditional models are defined. In the gfoRmula package, combined models are fitted across all time points, where time is usually included as a predictor. In the gFormulaMI package, separate models are fitted sequentially in time (equation (2)). Consequently, any differences in the results obtained between the two packages could be due to a combination of the different approaches to defining conditional models and the different approaches to handling missing data. To differentiate between these two sources of differences in the results, we also used both packages to analyse a complete dataset. For the complete data, we used the complete cases in the original data. For each analysis, we recorded the running time and estimated the Monte Carlo standard error for the treatment effect estimate (formulae for Monte Carlo standard errors are provided in Appendix D of the Supplemental material).

Four thousand, seven hundred fifty-nine individuals were eligible for inclusion in the analysis. Details on how the study sample was selected, baseline characteristics by treatment group, and amount of missing data by year, are provided in Section D, Tables 1 and 2, and Figure 1 of the Supplemental material. Of the 4,759 individuals included in the study, 2,255 were complete cases. Table 6 shows the results from each analysis along with the total running time.

**Table 6.** Results using data from the UK CF registry.

	Year 1	Year 2	Year 3	Year 4	Year 5
**Complete case data**					
gfoRmula (140.62 hours)					
TE	−0.18	−0.04	0.44	1.16	1.14
95% CI	(−0.79, 0.43)	(−0.91, 0.82)	(−0.74, 1.63)	(−0.61, 2.92)	(−0.76, 3.04)
MCSE	0.092	0.096	0.097	0.099	0.095
gFormulaImpute (0.50 hours)					
TE	−1.06	−1.08	0.20	1.05	0.92
95% CI	(−2.51, 0.39)	(−2.60, 0.44)	(−1.54, 1.95)	(−1.04, 3.14)	(−1.26, 3.11)
MCSE	0.023	0.024	0.028	0.033	0.035
**Partially observed dataset**					
gfoRmula (169.61 hours)					
TE	0.08	0.47	0.49	1.59	1.56
95% CI	(−0.55, 0.70)	(−0.39, 1.33)	(−0.79, 1.77)	(−0.35, 3.53)	(−0.47, 3.58)
MCSE	0.104	0.110	0.112	0.109	0.096
gFormulaImpute (11.14 hours)					
TE	0.39	−0.18	0.14	1.05	1.05
95% CI	(−0.57, 1.34)	(−1.29, 0.94)	(−1.01, 1.28)	(−0.29, 2.40)	(−0.33, 2.43)
MCSE	0.034	0.039	0.041	0.048	0.049

TE: treatment effect (estimated effects of adding hypertonic saline on FEV ${}_{1}$ % in people already taking DNase); CI: confidence interval; MCSE: Monte-Carlo standard error. Treatment effects for years 1–5 are estimated using two packages: gfoRmula and gFormulaImpute and using two datasets: Complete cases and the partially observed dataset. Times given in hours indicate computational time to run each analysis.

Overall, and in line with previous results,^20^ we found little evidence that adding hypertonic saline has any effect on FEV ${}_{1}$ % among individuals who are already established on DNase. All 95% confidence intervals contained 0, and the estimated effect sizes were not clinically significant. In the complete case data, estimates at years 3–5 were quite similar between gfoRmula and gFormulaMI, while the estimates at years 1 and 2 showed somewhat larger differences which were larger than the corresponding Monte-Carlo errors – these differences can be attributed to the different modelling assumptions made by the two implementations. Confidence intervals were wider from gFormulaMI, which is what one should expect since this fits separate models at each time point rather than assuming a common model as in gfoRmula. In the partially observed dataset, there were again differences in estimates larger than the Monte-Carlo errors, that can be attributed to a combination of the different way the two approaches handled missing data and also the different modellling assumptions made. Compared to the gfoRmula package, the gFormulaImpute package consistently obtained smaller Monte Carlo standard errors and had considerably shorter running time.

## 6. Discussion

G-formula via MI is an attractive approach for implementing parametric G-formula, that enables imputation of missing data and simulation of counterfactuals under the desired treatment regime(s) of interest. Moreover, it avoids the need to use bootstrapping for inference, which is particularly attractive in the context of combining MI for missing data with G-formula for causal inference. This is achieved by exploiting existing results for using MI to create synthetic datasets. The simulation results presented here suggest the G-formula via MI approach can perform well, requiring a relatively small number of imputations for reliable inference in the setup we used.

One alternative to imputing (actual) missing data when implementing G-formula is to fit each of the models required using the subset of records for which the variables involved in each model are fully observed, as is implemented for example in the gfoRmula package in R.^3^ These complete case model fits yield consistent estimates of the respective conditional model parameters provided the probability of having all the variables involved in the model is independent of the dependent variable conditional on the covariates.^23^ When the pattern of missingness in the longitudinal dataset is complex, consisting of both intermittent missingness and missingness due to dropout, such an assumption can sometimes be deemed more plausible than missing at random, whose meaning becomes complex in such settings.^24^ Thus an alternative possible version of the G-formula via MI approach when some data are missing is to fit each of the required models using their respective complete case fits. Such an approach would be more efficient and plausibly less likely to be biased than applying the method to the subset of individuals who have all variables at all time points fully observed.

To obtain point estimates and inferences with sufficiently small Monte-Carlo error, existing simulation based implementations of G-formula may require both the Monte-Carlo sample size to be large and also a large number of bootstrap samples to be used. Our simulations and data analysis suggest that reliable inferences can be obtained via MI methods using smaller Monte-Carlo sample sizes ( $n_{\text{syn}}$ ) and relatively few imputations (e.g. 50). Although implementation is relatively straightforward using existing MI packages, we have developed an R package gFormulaMI that interfaces with the mice package to perform the required data manipulation steps, estimate mean outcomes under each treatment regime of interest, and calculate the synthetic MI variance estimator. Imputation packages such as mice are flexible in regard model specification, for example allowing the possibility for the user to include interactions and higher order effects in models.

We note that while standard G-formula is typically implemented using Monte-Carlo simulation as we have described, an alternative version based on iterative conditional expectations can be used.^25^ This approach requires models for a series of conditional mean functions, rather than for the full distribution of the time-varying confounders and outcome. This makes it potentially less prone to model misspecification, particularly in the case of several time-varying confounders, where the standard approach requires a choice of factorisation to be made to specify the distribution of the time-varying confounders at each time point. Moreover, it does not require the use of simulation, and closed form variance estimators can be constructed based on estimating equation theory.^26^

In this paper we have focussed on G-formula where the outcome is a variable Y measured at one or, as in our CF example, multiple time points. G-formula can be used when the outcome is the time to some event of interest, for example based on discrete time logistic regression models.^4^ The G-formula via MI approach can also be used in this setting, by defining appropriate time-dependent binary indicators of survival.

Implementations of G-formula (e.g. the G-formula packages in Stata^2^ and R^3^) often fit models pooled across time points for each variable. This is achieved by formatting the data in so-called long form. Doing so permits borrowing of information across time points in the estimation of regression parameters, but of course relies on the validity of the assumption that the conditional distribution of confounders given earlier variables is homogeneous across time points. Although this approach could be implemented via the MI approach we have outlined, we do not believe it is possible using standard imputation software such as mice in R. This is because having transformed the data into long form, it is not (currently) possible to update values from one row of the data frame from another within the algorithm.

While our focus in this paper has been on static treatment regimes, G-formula can be used to estimate the effects of dynamic treatment regimes, where the exposure or treatment at a given time point is assigned dependent on the longitudinal history observed up to that time. The G-formula via MI approach can be extended to this case, by setting the treatment variables to missing in the augmented part of the dataset and then specifying how they should be imputed based on the preceding (in time) variables. This can be achieved for example in the mice package through the use of user specified deterministic (or indeed stochastic) imputation methods. Moreover, we believe the basic approach we have described can be extended and applied to more general and complex causal structures specified by a DAG.

## Supplemental Material

sj-pdf-1-smm-10.1177_09622802251316971 - Supplemental material for G-formula with multiple imputation for causal inference with incomplete data Supplemental material, sj-pdf-1-smm-10.1177_09622802251316971 for G-formula with multiple imputation for causal inference with incomplete data by Jonathan W Bartlett, Camila Olarte Parra, Emily Granger, Ruth H Keogh, Erik W van Zwet and Rhian M Daniel in Statistical Methods in Medical Research

## Data Availability

The gFormulaMI R package is available from CRAN. R code for the simulations and CF analysis are available from https://github.com/jwb133/gFormulaViaMultipleImputationPaper. Researchers can apply for access to the CF data from https://www.cysticfibrosis.org.uk/the-work-we-do/uk-cf-registry/apply-for-data-from-the-uk-cf-registry.
