# Supplementary material for: G-formula with multiple imputation for causal inference with incomplete data
Source: Stat Methods Med Res. 2025 Mar 31;34(6):1130–43. doi: 10.1177/09622802251316971 (PMC12209542; doi:10.1177/09622802251316971)
Supplement: sj-pdf-1-smm-10.1177_09622802251316971 - Supplemental material for G-formula with multiple imputation for causal inference with incomplete data [file sj-pdf-1-smm-10.1177_09622802251316971.pdf]

Supplementary material for ‘G-formula with multiple imputation for causal inference with incomplete data’ by Jonathan W. Bartlett, Camila Olarte Parra, Emily Granger, Ruth H. Keogh, Erik W. van Zwet, Rhian M. Daniel

Supplementary Appendix A

In this appendix we show that the variance estimator $\hat{V}_{\text{syn}}$ derived by Raghunthan *et al*[1] is consistent for the G-formula via MI estimator, using the results of Robins and Wang.[2]

No missing data

We first describe how the G-formula via MI estimator can be embedded into the setup of Robins and Wang,[2] considering the case first where there is no missing data in the original observed dataset. To that end, consider the augmented dataset where in the augmented part $\bar{L}$ and Y are set to missing and the treatment vector $\bar{A}$ is set to different values according to the treatment regimes of interest. Define the variable R that takes value 1 in the original observed data and 0 in the augmented part of the data. The full data vector is thus $F = (\bar{A}, \bar{L}, Y, R)$ and the observed vector is $O = (\bar{A}, \bar{L}R, YR, R)$.

The missing values in $\bar{L}$ and Y in the augmented part of the dataset are then multiply imputed. After imputation, in the G-formula via MI approach we estimate counterfactual means and contrasts of these using only data in the augmented rows, i.e. among those rows with $R = 0$. The complete data estimating functions are thus of the form

$$u(F, \beta) = (1 - R)W(\bar{A}, \bar{L}, Y, \beta),$$

where β is the parameter to be estimated. Suppose for example that potential outcomes are imputed for treatment regimes $\bar{a}_1$ and $\bar{a}_2$. Then letting $\beta = (\beta_1, \beta_2) = (E(Y^{\bar{a}_1}), E(Y^{\bar{a}_2}))$, the complete data estimating function could be of the form

$$u(F, \beta) = (1 - R)\left\{Y - \beta_1 I(\bar{A} = \bar{a}_1) - \beta_2 I(\bar{A} = \bar{a}_2)\right\} \begin{pmatrix} I(\bar{A} = \bar{a}_1) \\ I(\bar{A} = \bar{a}_2) \end{pmatrix}, \tag{1}$$

which corresponds to estimating the potential outcome mean for each of the two regimes by the sample means in the augmented data among those assigned to each regime.

Under the regularity conditions detailed by Robins and Wang,[2] the G-formula via MI estimator of β which uses $M = \infty$ imputations is asymptotically normal. The variance of the estimator with $M = \infty$ is given by equation A3 of Robins and Wang[2] as

$$\begin{aligned} \Sigma =& \tau^{-1}\Big[E\left\{U_{obs}(\psi^*, \beta^*)^{\otimes 2}\right\} + \kappa\Lambda(\psi^*)\kappa^T + \kappa E\left\{D(\psi^*)U(\psi^*, \beta^*)^T\right\} \\ &+ E\left\{D(\psi^*)U(\psi^*, \beta^*)^T\right\}^T \kappa^T\Big](\tau^T)^{-1}, \end{aligned}$$

where $A^{\otimes 2} = AA^T$. In the following we define the terms involved in the preceding expression, derive their values in our setting, and show that $\hat{V}_{\text{syn}}$ is a consistent estimator of this asymptotic variance. Parameters with a superscript $*$ denote the true value of the corresponding parameter.

The quantity τ is defined as

$$\tau = -E\left\{\frac{\partial U(\psi^*, \beta)}{\partial \beta^T}\right\}\bigg|_{\beta=\beta^*},$$

where

$$U(\psi, \beta) = u(F(\psi), \beta).$$

Here $F(\psi)$ denotes an imputed full data vector for a given random individual, with the imputation generated conditional on the value ψ of the imputation model parameter. Thus for an individual in the original data ($R = 1$), $F(\psi) = (\bar{A}, \bar{L}, Y, 1)$, while for an individual in the augmented part ($R = 0$), $F(\psi) = (\bar{A}, \bar{L}(\psi), Y(\psi), 0)$, where $(\bar{L}(\psi), Y(\psi))$ denotes the random imputed value drawn from $f(\bar{L}, Y|\bar{A}, \psi)$.

The quantity $U_{obs}(\psi, \mu)$ is defined as $E_\psi\{U(\psi, \beta)|O\}$. In our case, we have

$$\begin{aligned} U_{obs}(\psi, \beta) &= E_\psi\{U(\psi, \beta)|O\} \\ &= (1 - R)E_\psi\{W(\bar{A}, \bar{L}(\psi), Y(\psi), \beta)|\bar{A}, R = 0\} \\ &= 0, \end{aligned}$$

provided $E_\psi\{W(\bar{A}, \bar{L}(\psi), Y(\psi), \beta)|\bar{A}, R = 0\} = 0$. This holds for example for the complete data estimating function given in equation (1).

Letting $\hat{\psi}$ denote the observed data MLE of the imputation model parameters, $D(\psi)$ denotes the influence function of the estimator, and under standard regularity conditions $n^{1/2}(\hat{\psi} - \psi^*)$ is asymptotically normal with mean zero and covariance matrix equal to

$$\Lambda(\psi^*) = E\left\{D(\psi^*)^{\otimes 2}\right\} = I_{obs}^{-1} E\left\{S_{obs}^{\otimes 2}(\psi^*)\right\} I_{obs}^{-1}$$

where $S_{obs}(\psi)$ denotes the observed data score and I_{obs} the observed information matrix.

As noted by Robins and Wang following their Theorem 2, if, as we assume, the imputation model is correctly specified, $I_{obs} = E\left\{S_{obs}^{\otimes 2}(\psi^*)\right\}$, in which case $\Lambda(\psi^*) = I_{obs}^{-1}$.

Observations in the augmented dataset, with $R = 0$, do not contribute to the estimation of the imputation model, and so for such observations $D(\psi^*) = 0$. Conversely, observations in the original data with $R = 1$ do not contribute to the estimation of β in the imputed datasets:

$$U(\psi, \beta) = (1 - R)W(\bar{A}, \bar{L}, Y, \beta) = 0 \text{ if } R = 1$$

Consequently, $D(\psi^*)U(\psi^*, \mu^*) = 0$ for all observations, and so

$$E\left\{D(\psi^*)U(\psi^*, \beta^*)^T\right\} = 0.$$

Thus the asymptotic variance of the G-formula via MI estimator, with $M = \infty$, is given by

$$\Sigma = \tau^{-1}\kappa I_{obs}^{-1}\kappa^T(\tau^T)^{-1} \tag{2}$$

where

$$\kappa = E\{U(\psi^*, \beta^*)S_{mis}(\psi^*)^T\}, \quad S_{mis} = \frac{\partial}{\partial\psi}\log f(F|O;\psi)|_{\psi=\psi^*}.$$

Equation A1 from Robins and Wang gives that when imputations are generated from a Bayesian model (as in MI as originally conceived by Rubin), the (standardised by n) between imputation variance estimator $\bar{B}$ converges in probability as $m, n \to \infty$ to

$$\tau^{-1}\left\{\kappa I_{obs}^{-1}\kappa^T + E\left[\{U(\psi^*, \beta^*) - U_{obs}(\psi^*, \beta^*)\}^{\otimes 2}\right]\right\}(\tau^T)^{-1} = \tau^{-1}\left\{\kappa I_{obs}^{-1}\kappa^T + E\left[U(\psi^*, \beta^*)^{\otimes 2}\right]\right\}(\tau^T)^{-1},$$

since $U_{obs}(\psi^*, \beta^*) = 0$. Equation A2 gives that the (standardised) within-imputation variance

$\widehat{V}_\bullet$ converges to

$$\tau^{-1}E\left[U_{obs}(\psi^*,\beta^*)^{\otimes 2}+\{U(\psi^*,\beta^*)-U_{obs}(\psi^*,\beta^*)\}^{\otimes 2}\right](\tau^T)^{-1}=\tau^{-1}E\left[U(\psi^*,\beta^*)^{\otimes 2}\right](\tau^T)^{-1},$$

again using the fact $U_{obs}(\psi^*,\beta^*)=0$. Thus $\bar{B}-\widehat{V}_\bullet$ converges to

$$\tau^{-1}\kappa I_{obs}^{-1}\kappa^T(\tau^T)^{-1}=\Sigma$$

as required. Lastly, since in practice we can only implement the MI estimator with finite M, we must add an additional $M^{-1}\bar{B}$ to account for the additional Monte-Carlo variability, resulting in the variance estimator $\hat{V}_{\text{syn}}$.

Missing data

We now consider the extension to the case where there are some missing values in the original dataset. Now the observed vector is $O=(R,C,G(\bar{A},\bar{L},Y,C))$ where C indicates the missing data pattern and the function $G(\bar{A},\bar{L},Y,C)$ returns the variables observed under missingness pattern C. Then the missing data, which now consist of the missing values in the original data ($R=1$) and the missing potential outcomes in the augmented portion ($R=0$) are imputed from a Bayesian model jointly. Then the earlier proof for the setting of no missing data holds, with the key feature that $D(\psi^*)U(\psi^*,\mu^*)=0$ continuing to hold because the imputation model parameters are only estimated from the subset with $R=1$ while the complete data estimating function only depends on the augmented data with $R=0$. As such, the variance estimator $\hat{V}_{syn}$ remains valid.

Supplementary Appendix B

Recall that $\hat{B}$ is the between imputation variance of $\hat{\mu}_m$. Conditional on the observed data, the latter are normally distributed with mean $\bar{Y}$ and variance $\frac{\sigma^2}{n_{\text{obs}}} + \frac{\sigma^2}{n_{\text{syn}}}$. As such we have

$$\frac{(M-1)\hat{B}}{\frac{\sigma^2}{n_{\text{obs}}} + \frac{\sigma^2}{n_{\text{syn}}}} \sim \chi^2_{M-1}$$

Then the probability that $\hat{V}_{\text{syn}} < 0$ is given by

$$\begin{aligned} P\left\{(1+M^{-1})\hat{B} - \hat{V} < 0\right\} &= P\left\{(1+M^{-1})\hat{B} < \frac{\sigma^2}{n_{\text{syn}}}\right\} \\ &= P\left\{\frac{(M-1)\hat{B}}{\frac{\sigma^2}{n_{\text{obs}}} + \frac{\sigma^2}{n_{\text{syn}}}} < \frac{M-1}{(1+M^{-1})\left(\frac{\sigma^2}{n_{\text{obs}}} + \frac{\sigma^2}{n_{\text{syn}}}\right)}\frac{\sigma^2}{n_{\text{syn}}}\right\} \\ &= P\left\{\chi^2_{M-1} < \frac{M-1}{(1+M^{-1})\left(\frac{n_{\text{syn}}}{n_{\text{obs}}} + 1\right)}\right\} \end{aligned}$$

It follows that

$$P(\hat{V}_{\text{syn}} < 0) \approx P\left\{\chi^2_{M-1} < \frac{M}{\frac{n_{\text{syn}}}{n_{\text{obs}}} + 1}\right\}$$

From this expression it is clear that for a given value of M, as n_{syn} gets large this probability goes to zero. Alternatively, for a given value of n_{syn}, consideration of the normal approximation to the chi-squared distribution similarly shows the probability goes to zero as M increases.

Supplementary Appendix C

In the simulation study in the paper, data were generated from

$$
\begin{aligned}
&L_0 \sim N(0,1)\\
&P(A_0 = 1|L_0) = \text{expit}(L_0)\\
&L_1 \sim N(A_0 + L_0, 1)\\
&P(A_1 = 1|A_0, L_0, L_1) = \text{expit}(A_0 + L_1)\\
&L_2 \sim N(A_1 + L_1, 1)\\
&P(A_2 = 1|A_0, A_1, L_0, L_1, L_2) = \text{expit}(A_1 + L_2)\\
&Y \sim N(A_2 + L_2, 1)
\end{aligned}
$$

Supplementary Appendix D

In this appendix we provide additional details on the methodology and results from the illustrative example using UK CF Registry data.

Missing data were imputed using `mice` with 5 iterations. For both `mice` and `gFormulaMI`, the following model types were used: normal linear regression for continuous variables, logistic regression for binary variables, and polytomous regression for unordered categorical variables. When using `gfoRmula`, the conditional models for covariates and outcome included the full lagged history of all time-varying covariates (up to and including the 4th lag), all time-invariant covariates, and time as a categorical variable.

Formulae for the MCSE of the estimated treatment effects after k years are as follows:

$$\text{For estimates obtained using } \texttt{gfoRmula}\text{: } MCSE = \sqrt{\frac{\widehat{\text{Var}}(Y_k^{DN\&HS})}{n_{syn}} + \frac{\widehat{\text{Var}}(Y_k^{DN})}{n_{syn}}}$$

$$\text{For estimates obtained using } \texttt{gFormulaImpute}\text{: } MCSE = \sqrt{\frac{\hat{B}}{M}}$$

where $Y_k^{DN\&HS}$ and Y_k^{DN} are the simulated potential outcomes at k years under the two treatment strategies (DNase and hypertonic saline vs DNase only). $\hat{B}$ is the between-imputation variance and M is the number of imputations.

Supplementary Figure 1 shows how the study sample arose, Supplementary Table 1 provides details on the amount of missing data by year, and Supplementary Table 2 describes the baseline characteristics by treatment group.

Supplementary Table 1

$n = 4759$	Year 1	Year 2	Year 3	Year 4	Year 5
Missing data due to individuals leaving the study prematurely					
Left registry	0	262	311	312	302
Death	0	66	73	93	89
Censored	58	164	238	361	469
Missing data in baseline variables					
Sex	0	0	0	0	0
CFTR genotype	47	47	47	47	47
Ethnicity	31	31	31	31	31
Date of birth	0	0	0	0	0
FEV_1% decline	52	52	52	52	52
Missing data in time-varying variables					
FEV_1%	218	175	146	164	131
BMI z-score	79	72	75	43	50
IV days	0	0	0	0	0
IV hospital admissions	0	0	0	0	0
Pancreatic insufficiency	0	0	0	0	0
P. aeruginosa	4	10	11	6	5
Staphylococcus aureus	4	10	11	6	5
NTM infection	4	11	11	7	7

Amount of missing data by year in UK CF data analysis. Numbers missing data in time-varying variables in a given year is among those who were still in the study at that time. Left registry: administrative end of follow-up; censored: people were censored if they received an organ transplant, or initiated treatment with mannitol, ivacaftor, lumacaftor/ivacaftor or tezacaftor/ivacaftor; CFTR: Cystic Fibrosis Transmembrane Conductance Regulator; FEV_1% decline: Rate of decline in FEV_1% during the year prior to time 0; BMI: Body Mass Index; IV days: number of days on IV antibiotics since last review; IV hospital admissions: number of people with at least one IV hospital admission since the last review; NTM: Non-tuberculous mycobacterial

Supplementary Table 2

	Nil ($n = 232$)	HS ($n = 60$)	DN ($n = 4010$)	DN & HS ($n = 457$)
Female	117 (50.4%)	29 (48.3%)	2185 (54.5%)	220 (48.1%)
Age	24.3 (10.9)	19.5 (9.52)	21.1 (11.6)	18.7 (10.5)
Genotype				
High	160 (69.0%)	49 (81.7%)	3125 (77.9%)	376 (82.3%)
Low	23 (9.9%)	3 (5.0%)	308 (7.7%)	29 (6.3%)
None assigned	43 (18.5%)	8 (13.3%)	539 (13.4%)	49 (10.7%)
White	222 (95.7%)	59 (98.3%)	3832 (95.6%)	3 (0.7%)
IV days				
0	110 (47.4%)	26 (43.3%)	1754 (43.7%)	162 (35.4%)
1-14	36 (15.5%)	12 (20.0%)	754 (18.8%)	79 (17.3%)
15-28	27 (11.6%)	12 (20.0%)	516 (12.9%)	73 (16.0%)
28+	59 (25.4%)	10 (16.7%)	986 (24.6%)	143 (31.3%)
IV hospital admissions	96 (41.4%)	29 (48.3%)	1746 (43.5%)	238 (52.1%)
FEV_1%	65.9 (24.8)	66.2 (17.9)	69.9 (22.8)	67.7 (22.6)
FEV_1% decline	1.16 (1.76)	1.27 (1.64)	1.12 (1.53)	1.28 (1.55)
BMI z-score	-0.25 (1.26)	-0.29 (1.04)	-0.07 (1.14)	-0.21 (1.13)
P. aeruginosa	153 (65.9%)	39 (65.0%)	2420 (60.3%)	275 (60.2%)
Staphylococcus aureus	101 (43.5%)	22 (36.7%)	1614 (40.2%)	190 (41.6%)
NTM	12 (5.2%)	5 (8.3%)	183 (4.6%)	27 (5.9%)
Pancreatic insufficiency	198 (85.3%)	50 (83.3%)	3548 (88.5%)	420 (91.9%)

Summary of characteristics by treatment combination observed in the first year of follow-up in UK CF data analysis. Continuous variables are summarised using mean (standard deviation (SD)) and categorical variables are summarised using numbers (%). Nil: Drop DNase and do not start hypertonic saline; HS: Drop DNase and start hypertonic saline; DN: Continue DNase and do not start hypertonic saline; DN&HS: Continue DNase and start hypertonic saline. FEV_1% decline: Rate of decline in FEV_1% during the year prior to time 0; BMI: Body Mass Index; IV days: number of days on IV antibiotics since last review; IV hospital admissions: number of people with at least one IV hospital admission since the last review; NTM: Non-tuberculous mycobacterial

Supplementary Figure 1

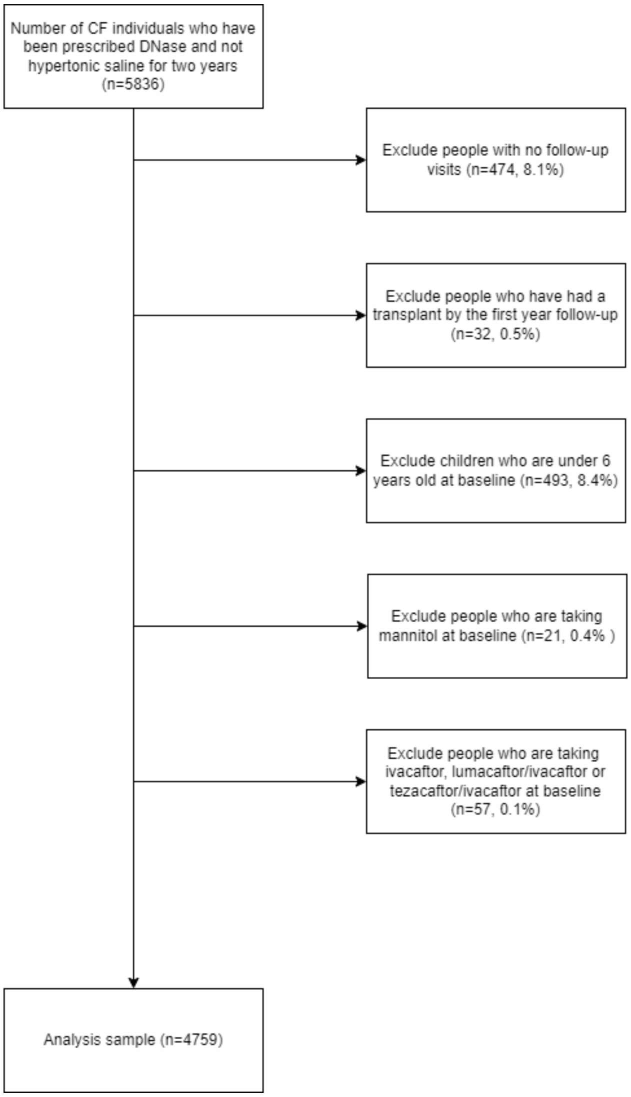

Flowchart of participant selection into the study sample for UK CF data analysis.

References

[1] Trivellore E Raghunathan, Jerome P Reiter, and Donald B Rubin. Multiple imputation for statistical disclosure limitation. *Journal of Official Statistics*, 19(1):1, 2003.

[2] J M Robins and N Wang. Inference for imputation estimators. *Biometrika*, 85:113–124, 2000.
